# Viewpoint: Personalizing Statin Therapy

**DOI:** 10.5041/RMMJ.10108

**Published:** 2013-04-30

**Authors:** Shlomo Keidar, Aviva Gamliel-Lazarovich

**Affiliations:** 1 Internal Medicine A, The Rappaport Family Institute for Research in the Medical Sciences, Rappaport Faculty of Medicine, Technion-Israel Institute of Technology and Rambam Medical Center, Haifa, Israel; and; 2 Lipid Research Laboratory, The Rappaport Family Institute for Research in the Medical Sciences, Rappaport Faculty of Medicine, Technion-Israel Institute of Technology and Rambam Medical Center, Haifa, Israel

**Keywords:** Framingham Risk Score, cardiovascular disease, coronary artery calcification, carotid artery intima-media thickness, statin, atherosclerosis

## Abstract

Cardiovascular disease (CVD), associated with vascular atherosclerosis, is the major cause of death in Western societies. Current risk estimation tools, such as Framingham Risk Score (FRS), based on evaluation of multiple standard risk factors, are limited in assessment of individual risk. The majority (about 70%) of the general population is classified as low FRS where the individual risk for CVD is often underestimated but, on the other hand, cholesterol lowering with statin is often excessively administered. Adverse effects of statin therapy, such as muscle pain, affect a large proportion of the treated patients and have a significant influence on their quality of life.

Coronary artery calcification (CAC), as assessed by computed tomography, carotid artery intima-media thickness (CIMT), and especially presence of plaques as assessed by B-mode ultrasound are directly correlated with increased risk for cardiovascular events and provide accurate and relevant information for individual risk assessment. Absence of vascular pathology as assessed by these imaging methods has a very high negative predictive value and therefore could be used as a method to reduce significantly the number of subjects who, in our opinion, would not benefit from statins and only suffer from their side-effects.

In summary, we suggest that in very-low-risk subjects, with the exception of subjects with low FRS with a family history of coronary artery disease (CAD) at young age, if vascular imaging shows no CAC or normal CIMT without plaques, statin treatment need not be administered.

Cardiovascular (CV) events, associated with vascular atherosclerosis, are the major cause of death in Western societies. The pathogenesis of atherosclerosis involves inflammation and cholesterol accumulation in the vascular wall, leading to plaque formation and progression which further leads to clinical manifestation of ischemic heart disease and cerebrovascular events. Since atherosclerosis is a chronic progressive vascular illness, improved risk assessment has clinical importance in terms of identifying atherosclerotic changes at an early stage when therapeutic intervention can still improve the prognosis.

Current risk estimation tools, such as Framingham Risk Score (FRS), are statistics-based tools which employ standard multiple risk factors such as age, sex, smoking, blood pressure, serum metabolic components, etc. According to FRS, the majority (about 70%) of the general population is asymptomatic and will have a less than 10% risk of experiencing CV events in the next 10 years. On the other hand, a substantial number of CV events will occur in these low- to medium-risk subjects.^1^,^2^ Thus, FRS alone is limited in predicting which of these asymptomatic people will eventually experience a cardiovascular event.

Based on FRS, and according to the guidelines, high-risk patients, with an estimated 10 years event rate higher than 20%, are referred to statin treatment as primary prevention, whereas medium-risk (10%–20%) or low-risk (less than 10%) patients might not be eligible for treatment with statins for primary prevention.^2^,^3^

Thus, two issues need to be discussed: how can we improve individual risk assessment and how can we achieve better prevention?

Lipid burden is known to play a major role in atherosclerosis lesion progression.^4^ Therefore, lowering circulating cholesterol levels became an important target in reducing cardiovascular events, and, indeed, secondary prevention by statin therapy was shown in many clinical trials to be associated with reduced morbidity and mortality and higher survival rates. However, the evidence for efficacy of statins in mortality prevention among patients without a history of cardiovascular disease is controversial. Whereas some meta-analyses^5^,^6^ reported reduction in all-cause mortality, another study did not find evidence for the benefit of statin therapy in primary prevention.^7^ The inclusion of low- to medium-risk subjects, who have lower probability for atherosclerosis manifestation, might contribute to increasing the real number needed to treat (NNT) and as a result reduced statins’ absolute efficacy in some of the studies.^8^

Side-effects of statin therapy vary, and a significantly increased rate of new-onset diabetes^9^ is among the observed adverse events. But the main complaint affecting 10%–20% of patients is muscle pain, which has a significant influence on quality of life and often results in reduced therapy compliance.^10^ Therefore, exposure of healthy subjects to lifelong statin therapy needs clear and solid evidence for benefits which outweigh the adverse events.

Considerable efforts have been made in recent years to characterize additional atherogenic factors, which combined with FRS will improve the risk assessment accuracy. However, evaluation of a variety of factors claimed to improve prediction beyond FRS are still controversial and have not added significant value to risk assessment,^11^ proving the need for better-quality markers. As atherosclerosis is characterized by abnormality of the vascular wall, the immediate tool for identifying subclinical atherosclerosis is arterial imaging.

A growing body of evidence points to the advantages of two non-invasive imaging techniques, which provide accurate and relevant information for individual risk assessment.

Coronary calcium score (CCS), as assessed by computed tomography (CT), allows identification and quantification of vascular plaque burden. The results of meta-analysis in many clinical studies applying this technique demonstrate that 40%–50% of asymptomatic patients had zero CCS and an extremely low annual cardiovascular events rate.^12^ Absence of coronary artery calcification (CAC) had a very high negative predictive value (>98%), with a 5-year follow-up, making preventive intervention redundant in many asymptomatic subjects. New data from the Multi-Ethnic Study of Atherosclerosis (MESA) by Nasir et al.,^13^ which was presented in the last American Heart Association symposium, showed that in the absence of CAC, 537 subjects with a FRS of less than 10% and a mean age of 62 years would be treated for 5 years to prevent *only a single* cardiovascular event! Thus, not only will the majority of patients not benefit from the treatment, but the well-being of more than 50 subjects could be significantly affected if some 10% of these patients suffer from side-effects of statins.

The major drawback of CT vascular imaging is the added risk of radiation exposure. However, the newer CT equipment produces relatively low radiation doses (less than 1 mSv), which makes the benefits of the additional information gained by CAC imaging outweigh the risks of radiation.

It is important to note that, in our view, the use of cardiac CT angiography (CCTA) to rule out coronary disease is not recommended, because incremental information gained by this method, compared to CAC, does not merit the higher radiation and costs.

Carotid B-mode ultrasound imaging provides another non-invasive modality for the detection of arterial vascular pathology. Increased carotid artery intima-media thickness (CIMT) and especially presence of plaques are associated with an increased risk of cardiovascular events. Recent studies have shown that carotid ultrasound might identify subclinical atherosclerosis earlier than CAC.^14^ Given the progressive nature of atherosclerosis, carotid ultrasound might provide a clinical decision-making tool for earlier or aggressive preventive therapy intervention and possible improved outcomes. It is important to stress that a major limitation to this examination is that it should only be performed by experienced operators.

Other non-invasive procedures, which are frequently done in low-risk subjects, such as stress tests with or without thallium, are not justified in our opinion because they will show significant coronary disease only when obstruction of the artery is greater than 70%. Beyond the low sensitivity of these methods, thallium stress tests also involve radiation, and these tests are costly.

Although the rate of atherosclerosis progression in patients with very low risk was not shown in previous studies, repeated examinations to follow the vascular atherosclerotic process might be needed. At this point we suggest repeating the vascular imaging examination every 5 years, which is about the mean follow-up in previous randomized clinical studies, during which no major cardiovascular event occurred in these very-low-risk patients. Further prospective studies are required to determine if and when repeated examination is required, but this time-frame is reasonable in light of the position stated by the panel of radiologists who recommended that patients who have normal carotid ultrasound (US) studies but marked risk factors, thus not low risk, might be evaluated every 3–5 years.^15^

In Israel, many thousands of subjects with very low FRS (less than 6%), mostly women, are treated with statins (personal knowledge). In a recently published study,^16^ half of low-risk patients who underwent CCTA showed no vascular pathology. Thus, a large proportion of subjects from this category will be treated with statins, and, of these subjects, at least 10%–15% suffer from myopathy but are still encouraged by their physicians to continue the medication. We suggest that in these very-low-risk subjects, if vascular imaging shows no CAC or normal CIMT without plaques, statin treatment need not be administered, with the exception of subjects with low FRS with a family history of CAD at young age.

In our view, vascular imaging is also cost-effective, especially in the long run. The cost of CT for evaluation of CAC or US carotid artery examination in our institution is about $130, which is about the equivalent of 1 year of treatment with low-dose generic statins, blood tests, and visits to the physician.

In summary, we suggest using vascular imaging as a method to reduce significantly the number of subjects who, in our opinion, would not benefit from statins and only suffer from their side-effects. As P.K. Shah has previously well formulated^17^: “If the goal of using a statin is to reduce atherothrombotic cardiovascular events, then it is unrealistic to expect those patients without significant atherosclerosis to benefit from statin therapy even if they have hyperlipidemia. In such subjects, one can only expect side effects.”

## Abbreviations:

CAC: coronary artery calcification;
CAD: coronary artery disease;
CCTA: cardiac CT angiography;
CIMT: carotid artery intima-media thickness;
CV: cardiovascular;
CVD: cardiovascular disease;
FRS: Framingham Risk Score;
US: ultrasound.
